# Annotations

**Published:** 1900-09-22

**Authors:** 


### Annotations.

### Rate-supported Hospitals.

One outcome of the differences which have existed for some time in regard to the management of the Aberdeen Royal Infirmary has been the formulation of a demand on the part of certain advanced advocates of municipalisation—as socialism is now spelled—that the support of the infirmary should be thrown upon the rates. A resolution has been passed by a meeting of citizens to the effect that "the only way of making adequate provision for the sick is by having the management of the infirmary placed in the hands of some popularly-elected board, with statutory rating powers at its disposal." We may be allowed to doubt whether the good people of Aberdeen quite know what they are asking for when they put the matter in this, apparently, simple form. What is quite certain is that rate support and private charity are incompatible, and that anything which should subject charity hospitals to municipal control must at once alienate the gifts of those upon whose support they have hitherto depended. What a man receives from the State, the municipality, or the guardians of the poor, he receives as a right; and directly these bodies begin to "give" what is not justly due they embark upon a career of jobbery and corruption. What a man receives from a charity hospital he receives as a free gift; he has no rights in the matter at all, and herein must always lie the great distinction between these two forms of medical relief. We make bold to say that among the various forms taken by charity that of giving relief to the poor in sickness will always occupy a prominent position, and that if to-morrow all the hospitals were taken over by the State and devoted to the treatment of those who could show some claim or right to the benefits they would dispense, other hospitals would soon spring up, founded by charitable people, to afford assistance to those who would be left out by the necessarily strict rules in accordance with which public money must always be distributed. The fact is that we already have State hospitals. The hospitals of the State are the workhouse infirmaries, to which all contribute and to which all have a right of entry if they should become unable to obtain medical assistance in any other way. Let those who would municipalise the hospitals turn their attention to improving the workhouse infirmaries. They may be quite sure that, however thoroughly they reform these institutions there will always be a large number of deserving people to whom the charitable will be glad to hold out a helping hand by the aid of the charity hospitals. Not necessarily the very poor, who are already provided for, nor the well-to-do, who can, if they choose to pay for it, get the best advice, but people who can be saved from the workhouse by a little help, or can be saved from permanent illness by a little timely treatment, and yet have neither the money with which to pay for these things, nor yet the absolute poverty which would obtain them as a right. We do not deny that there is room for State hospitals in England. We already have them, and they might in many instances be much improved. But they by no means take the place, and never can take the place of charity hospitals, the very essence of whose work is not, as some people would have it, that they give to those who have a claim, but that out of pure charity they give to those who have no claim at all. It is in their power to do this in a manner which could not be allowed for a moment to any State institution distributing public funds, and it is this power of giving help without raising any question of rights or of claims that makes the charity hospital, supported by voluntary contributions, occupy a position and fill up a gap in English social life which can never be occupied by rate-supported institutions.

### Insurance Certificates.

The attempts that are being made by insurance companies to cut down the fees for medical certificates, if read parallel with much that has lately been made public in regard to the commissions given by such offices to those by whom "business" is introduced, should make people very wary about life insurance. No doubt the managers of these companies have learned by experience that the lack of union which is so manifest among medical men renders them an easy prey; but we would point out the extremely undesirable position in which a medical man places himself when he accepts the lower rates which are now being offered. When a company which offers a fee of a guinea for an examination in cases in which the sum insured is over £250, gives only half that fee in cases of insurance for smaller amounts, one may well ask whether they mean that in the latter case they only require an imperfect examination. What is sufficiently obvious to all who are accustomed to careful clinical work is that every year increases the complexity of the problem involved in insurance work, and that every advance in our knowledge of disease seriously adds to the responsibility of signing a certificate on anything short of a complete examination. Instead of the fees for such certificates being reduced, they ought to be materially increased; and they would be increased if the profession had within it even the elements of business capacity or power of combination. What are called the "dictates of humanity" have often stood in the way of combinations to raise fees, and the pressure of public opinion has pulled many a doctor out of bed on cold and stormy nights, the miserable man going braving the elements, without hope of fee, lest, perchance, life might be lost from his "inhumanity" in refusing to go. But in dealing with these insurance companies there is no question of humanity. The whole affair is a mere business transaction, in regard to which, seeing that the doctors have the companies in the hollow of their hands, they may

ANNOTATIONS—*continued.*

fairly and appropriately combine to raise prices instead of allowing themselves to be beaten down. And yet we find the beating down process going on uninterruptedly. So much for medical combination! How are we to expect sick clubs and friendly societies, in regard to which the question of humanity really does sometimes arise, to be brought to reason, when insurance societies are permitted to fix their own prices for medical certificates, and to pay for a complete examination and professional opinion no more than they would pay to an ordinary chemist for an examination of the urine alone?

### The Teeth of the Rising Generation.

Among the various branches of science teaching which are undertaken in our elementary schools it might be well if room could be found for some very definite and dogmatic instruction in regard to the importance of the teeth, and the necessity of carefully preserving such masticatory machinery as the vagaries of heredity may have handed down to us. It is all very well to teach children about the function of their livers and the action of their lungs. Poor little brats! what can they do if their livers do go wrong? and how can they help themselves even if they do get bronchitis? But for their teeth they can do much, and what they can do is just what no one else can do for them. Dr. Richard Grady, of Baltimore, dentist to the United States Naval Academy, hangs up in his office the motto, "Good Teeth, Good Health," and to it he appends the following sententious aphorisms: "Without good teeth there cannot be thorough *mastication*. Without thorough mastication there cannot be perfect *digestion*. Without perfect digestion there cannot be proper *assimilation*. Without proper assimilation there cannot be *nutrition*. Without nutrition there cannot be *health*. Without health what is *life*? Hence the paramount importance of the teeth." Nothing could be neater. One thing follows the other as inevitably as in the story of "The House that Jack Built," and it is to be hoped that the boys in the United States Navy profit by the lesson so plainly laid before them. We can well imagine, however, that if these maxims were displayed in our Board Schools it would not be long before our boys picked out the weak point underlying all such advice. "If," they would say, "you really believe all this, why on earth do you not send a dentist to look after these teeth, which, as you say, are so essential to our health, and to the well-being of the nation?" And it must be confessed that the answer is not at once apparent. In the meantime, in teaching each child to look after his own teeth, practice might be made to follow close on the heels of theory, and the prizes might go not to the boy who could most glibly tell us all about dentine and enamel, but to the one who showed the cleanest set of teeth.

### A Municipal Sanitary Missionary.

The Vestry of St. George the Martyr, Southwark, are a curious mixture of the progressive and the obstructive. One moment we hear of them as at daggers drawn with their medical officer of health in regard to some very obvious sanitary requirement, and the next as going even in advance of the requirements of the law in regard to sanitary inspection. Just now they are all for appointing a third female sanitary inspector to visit the houses of the poor and give advice in sanitary matters. The difficulty is that the title "sanitary inspector" is already ear-marked by the Public Health Act, by which also the duties of the office are laid down, so that it is impossible for the vestry to do quite as they like in the matter. The Local Government Board seems, however, to have stated that their sanction would not be required to the appointment of an officer under any other title, whom the vestry might be legally empowered to appoint, to carry out the duties proposed. The vestry have therefore decided to dub the additional inspector "lady sanitary officer and health inspector," under which title she will no doubt be able to find plenty of work to do. She will, of course, have to recognise that she is not a sanitary inspector; it is probable that she will have none of that official right of entry which a fully-fledged sanitary inspector possesses; and no doubt a good deal of care will have to be exercised in the apportioning of her work to prevent such excessive and repeated visitations as might make inspection stink in the nostrils of the inhabitants of the district. But if care be taken in this regard, and if the woman appointed be of the right sort—one who will work harmoniously with the medical officer and with other sanitary officials—we can hardly doubt that good will result. The very essence of the appointment is that the person appointed shall be a nurse of experience, and that she shall be a teacher rather than an inspector, a guide rather than a spy; and we can hardly doubt that one great difficulty of the position will lie in this, that at the end of the year she will have nothing to show. The more quietly and unobtrusively she spends her time sowing the good seed of sanitary teaching in regard to drains and smells, to fresh air and the care of infants, the less will she have it in her power to make a parade of what she has done. If the new officer comes across anything that clearly ought to be rectified by landlord or by sanitary authority, no doubt she will report it, but her main work must be that of a municipal sanitary missionary.

### Is Copper a Poison?

In a paper read before the British Association on the Effects of Copper on the Human Body, Dr. Hime, of Bradford, related some investigations which he had made, with the object of ascertaining the effects of copper when swallowed, or when absorbed either by swallowing or in other ways. With this object the excretions had been examined for a period of several months from a number of healthy persons engaged, some for many years, in dealing with copper, either in smelting works or as workers in its alloys, brass, &c., and from healthy persons unconnected with any kind of copper work who had intentionally swallowed some compound of copper. Copper was found in relative abundance in the excretions of all these persons, yet they enjoyed perfect health, and the conclusion he arrived at was that much that had been said in regard to the injurious effects of this metal was without foundation in fact. He went on to draw various conclusions as to the justifiability of using copper, with the object of improving the appearance of preserved peas, &c., maintaining that in such cases "the people who eat 'coppered' vegetable do not consume 'copper.' The chemical compound of copper they swallow is not copper at all, and they are not injured." This is an argument that does not appeal to us. The point of interest which he makes is that certain people sufficiently charged with copper to show it in their excretions enjoy perfect health.

